# Transcriptomic Characterization of Adventitious Root Formation in *Cunninghamia lanceolata* (Lamb.) Hook. During Cutting Propagation

**DOI:** 10.3390/genes17091008

**Published:** 2026-08-26

**Authors:** Yuting Wei, Ziyi Wang, Zezhong Lin, Liming Zhu, Zhaodong Hao, Shunde Su, Shuijin Luo, Xiaoli Jiang, Ling Ye, Yuhan Zhang, Lingfeng Yu, Jinhui Chen, Renhua Zheng

**Affiliations:** 1 State Key Laboratory of Tree Genetics and Breeding, Co-Innovation Center for Sustainable Forestry in Southern China, Nanjing Forestry University, Longpan Road 159, Nanjing 210037, China; yutingwei2001@163.com (Y.W.); 18851681796@163.com (Z.W.);; 2 Fujian Provincial Key Laboratory of Forest Cultivation and Forest Products Processing and Utilization, Fujian Academy of Forestry Sciences, Fuzhou 350012, China; aganlzz@126.com (Z.L.); zhulm777@163.com (L.Z.);; 3 Jiangle State-Owned Forest Farm of Fujian Province, Sanming 353300, China13960590626@163.com (X.J.)

**Keywords:** *C. lanceolata*, adventitious root formation in cuttings, transcriptome, phytohormone signal transduction

## Abstract

**Background**: *Cunninghamia lanceolata* (Lamb.) Hook. (*C. lanceolata*) is an important timber tree species in southern China. However, the molecular regulatory mechanisms underlying adventitious root formation during cutting propagation remain largely unclear. The lack of genetic resources has hindered molecular breeding efforts in this species. **Methods**: In this study, transcriptome analysis was performed on the root systems of scions from elite *C. lanceolata* clones at 7, 30, and 60 d after cutting. **Results**: Approximately 69.30 Gb of clean data were obtained. De novo assembly and gene prediction yielded 43,433 protein-coding genes, of which 32,886 (75.7%) were functionally annotated. Temporal clustering and comparative functional enrichment analyses revealed a distinct temporal functional shift during adventitious root development in *C. lanceolata*. Early stages were dominated by metabolic processes such as pyrimidine metabolism and carbohydrate biosynthesis, whereas later stages were governed by phytohormone signal transduction. Key components of the auxin pathway, including *AUX1*, *AFB*, *IAA*, and *SAUR*, exhibited dynamic and differential expression, which may be associated with adventitious root growth. Based on the transcriptome data, we identified nine members of the *PIN* gene family. Both transcriptomic expression profiles and qRT-PCR validation demonstrated that these *PIN* genes showed divergent expression trends across developmental stages, suggesting that they may participate in rooting by regulating polar auxin transport. **Conclusions**: This study systematically elucidates the molecular network underlying adventitious root formation in *C. lanceolata* cuttings. It enriches the omics resources for conifers and provides a theoretical foundation and omics basis for molecular breeding and efficient propagation of elite *C. lanceolata* clones.

## 1. Introduction

*C. lanceolata* is an important timber tree species endemic to southern China [1,2]. It exhibits rapid growth, excellent wood properties, and high economic value. It is widely distributed across 17 provinces in southern China [3] and plays a dominant role in the country’s timber supply and forestry economy [4]. However, increasing demand for high-quality planting stock in forestry management poses serious challenges to the large-scale propagation of elite *C. lanceolata* clones. Cutting propagation is a common technique for clonal propagation of woody plants, which can effectively preserve the superior genetic traits of donor plants. Nevertheless, the scions of *C. lanceolata* lack root primordia before cutting [5]; adventitious root formation therefore depends entirely on the induction of root primordia through complex molecular regulatory networks after cutting [6]. Thus, elucidating the molecular mechanisms underlying adventitious root formation in *C. lanceolata* is of great significance for overcoming bottlenecks in elite clone propagation and for advancing molecular breeding efforts in this species.

Adventitious root formation is a strictly timed and regulated developmental event. It typically proceeds through three sequential stages: induction, initiation, and elongation. Phytohormones play an indispensable and central regulatory role in this process. Among them, auxin is recognized as the most critical hormonal signal that promotes adventitious root formation. It activates downstream signaling cascades to initiate root primordium formation. Asymmetric auxin distribution in planta is key to initiating adventitious rooting. This spatial distribution pattern relies primarily on the precise regulation of the polar auxin transport system [7]. PIN-FORMED (*PIN*) family proteins function as auxin efflux carriers. Through their asymmetric polar localization on the plasma membrane, they mediate directional auxin efflux between cells. This establishes auxin concentration gradients and local auxin maxima in specific tissue regions [8]. In recent years, significant progress has been made in understanding the functions of *PIN* genes in adventitious root formation. In *Malus domestica*, *MdPIN* family genes exhibit dynamic and differential expression patterns throughout adventitious root development. *MdPIN8* and *MdPIN10* were significantly upregulated during the induction stage, and all *MdPIN*s were upregulated during the initiation stage [9]. In *Oryza sativa*, *OsSEC3A* regulates adventitious root development by mediating the intracellular trafficking of *OsPIN1b* [10]. Collectively, these cross-species findings consistently demonstrate that the *PIN* gene family plays an irreplaceable role in adventitious root formation.

Current studies on the functions of *PIN* genes in adventitious root formation have mainly focused on herbaceous plants and a few horticultural crops. However, systematic research on their distribution and functions in conifers such as *C. lanceolata* remains lacking. To address this knowledge gap, we combined time-series transcriptome sequencing and qRT-PCR to systematically characterize the gene expression network during adventitious root formation in *C. lanceolata* cuttings. We particularly focused on phytohormone signal transduction, a core regulatory pathway. Furthermore, we comprehensively identified and phylogenetically analyzed the *PIN* gene family in *C. lanceolata*. We further explored their dynamic expression patterns across different stages of adventitious root development, as well as their potential roles in polar auxin transport and hormone signaling networks. This study not only fills a gap in *PIN* gene research in *C. lanceolata*, but also enhances our understanding of the molecular mechanisms underlying adventitious root formation in conifer species.

## 2. Materials and Methods

### 2.1. Plant Materials

The experimental material used in this study was the elite *C. lanceolata* clone ‘Minshan No. 1’, obtained from the National Improved Variety Base of Jiangle State-Owned Forest Farm in Fujian Province, China. New shoots were collected from healthy, pest-free mother plants and used as cuttings. Cuttings were grown under field experiment conditions with 60–80% relative humidity and natural light. Samples were collected at 7, 30, and 60 d after cutting [11,12]. The basal portion of the stem, approximately 3–5 cm above the cutting base, was harvested at each sampling time point for subsequent analysis. Three biological replicates were set for each time point. All samples were snap-frozen in liquid nitrogen and stored at –80 °C until RNA extraction.

### 2.2. RNA Extraction and Transcriptome Sequencing

Total RNA was extracted from basal stem tissues using the RNA Pure Kit (TIANGEN, Beijing, China). RNA integrity, purity, and concentration were assessed using NanoDrop (Thermo Fisher, Waltham, MA, USA), and an Agilent 2100 (Agilent Technologies, Santa Clara, CA, USA). For library construction, mRNA was enriched using Oligo(dT)-coated magnetic beads, and sequencing libraries were prepared using the Hieff NGS^®^ Ultima Dual-mode mRNA Library Prep Kit (Yeasen, Shanghai, China) following the manufacturer’s instructions. The libraries were constructed as non-strand-specific, with an average insert size of approximately 150 bp. Following library construction, qualified libraries were sequenced on the Illumina NovaSeq 6000 platform (Illumina, Inc., San Diego, CA, USA) using paired-end 150 bp.

### 2.3. Transcriptome Analysis

Raw reads were subjected to quality filtering using fastp (v0.20.1), with key parameters set as -q 20 -l 100 -n 10. Reads containing adapters were removed. Reads with more than 10% N bases were discarded. Reads with more than 50% of bases having a quality score (Q) ≤ 10 were also filtered out. After quality control, the remaining high-quality reads were designated as Clean Data.

For transcriptome assembly, all clean reads from all samples were pooled into a single input dataset, and a unified reference transcriptome was assembled using Trinity (v2.6.6) [13]. A Python (v3.7) script was used to extract the longest transcript as the non-redundant gene set, and the final non-redundant gene sequences were obtained and stored in FASTA format.

Clean reads from each sample were independently mapped back to the above non-redundant reference transcriptome using HISAT2 (v2.0.4), and transcript quantification was performed using StringTie (v1.3.4d). Both read counts and FPKM values were generated.

### 2.4. Functional Annotation

Functional annotation was performed using the Non-Redundant Nucleotide Database (Nr), the Protein Families database (Pfam), Swiss-Prot, Gene Ontology (GO), and the Kyoto Encyclopedia of Genes and Genomes (KEGG).

### 2.5. Analysis of Differential Expressed Genes

Differential expression analysis was performed on the read count data using edgeR (v3.34.1) [14]. Specifically, an ANOVA-like test was conducted using the glmQLFTest function to identify genes with significant expression changes across all samples; on this basis, pairwise comparisons were further performed between different time points to identify stage-specific differentially expressed genes. Differentially expressed genes were defined as those with |log2 fold-change| > 1 and FDR < 0.05. GO and KEGG enrichment analyses were performed using clusterProfiler (v4.0.5) [15] with the parameters *p*-valueCutoff = 0.05 and *q*-valueCutoff = 0.2; multiple testing correction was applied using the Benjamini–Hochberg method. Gene expression patterns were visualized using pheatmap (v1.0.12) with complete linkage clustering.

### 2.6. PIN Gene Family Identification and Sequence Analysis

Based on the hidden Markov model of the PIN domain (PF03547) from the Pfam database, we used HMMER (v3.10) to search for candidate genes in the *C. lanceolata* Unigene protein database. Meanwhile, we performed local BLASTP searches using eight *Arabidopsis thaliana* (*A. thaliana*) AtPIN protein sequences as queries, with an E-value threshold of 1 × 10^−5^ and all other parameters set to default [16]. The overlapping candidates from both methods were retained. These candidates were further validated using SMART (http://smart.embl-heidelberg.de/ accessed on 20 June 2026) and NCBI CDD (https://www.ncbi.nlm.nih.gov/Structure/cdd/wrpsb.cgi accessed on 20 June 2026) to confirm the presence of conserved domains [17].

### 2.7. Phylogenetic Analysis

To investigate the phylogenetic relationship between *C. lanceolata* PIN proteins and *A. thaliana* PIN proteins, we downloaded the *A. thaliana* PIN protein sequences from the Phytozome database. Multiple sequence alignment of *C. lanceolata* PIN proteins and *A. thaliana* PIN proteins was performed using MAFFT [18]. The software automatically selected the optimal alignment strategy (FFT-NS-2) based on the sequence data; for high-precision alignment, the L-INS-i algorithm was additionally applied with parameters -localpair and -maxiterate 1000. The alignment results were refined using Trimal (v1.2) [19] with the -automated1 parameter for automatic trimming. A maximum-likelihood phylogenetic tree was constructed using IQ-TREE (v2.0) [20]. The best-fitting substitution model was automatically determined by Model Finder (with the -m TEST option). Branch support was assessed using the Ultrafast Bootstrap method with 1000 replicates (-bb 1000), and the number of CPU cores was set to -nt AUTO. The resulting tree was visualized and annotated using the online interactive tool iTOL (v6.7.4, https://itol.embl.de. accessed on 25 June 2026) [21,22].

### 2.8. qRT-PCR Analyses

Gene-specific primers were designed using Primer Premier (v3.0) [23]. The Actin gene of *C. lanceolata* was used as the internal reference gene [24]. Total RNA from each sample was reverse-transcribed into cDNA using a reverse-transcription kit (Vazyme, Nanjing, China). qRT-PCR was performed with SYBR Green Master Mix (Vazyme, Nanjing, China) on a real-time PCR system (Applied Biosystems, Foster City, CA, USA). Three biological replicates were set for each sample. Relative expression levels of target genes were calculated using the 2^−ΔΔCT^ method [25].

## 3. Results

### 3.1. Overview of Transcriptome Sequencing Data Quality

To investigate transcriptomic changes during root development in *C. lanceolata*, we performed RNA-seq on samples collected at three time points (7, 30, and 60 d after cutting; designated C2-1, C2-2, and C2-3) and observed morphological changes (Figure S1). Each sample yielded between 39,954,166 and 66,105,062 raw reads. After quality control, each sample generated 5.9 to 9.9 Gb of clean data. The Q30 values ranged from 97.88% to 98.15%, and the GC content ranged from 42.91% to 43.11%, indicating high data quality (Table 1).

High-quality clean reads were assembled de novo, yielding 542,161 transcripts (Table 2). After removing redundancy, a total of 469,733 unigenes were obtained. Among them, 342,358 unigenes ranged from 200 to 500 bp in length; 73,287 were 500–1000 bp; 31,092 were 1000–2000 bp; and 22,996 unigenes exceeded 2000 bp. A total of 43,433 coding sequences were predicted (Table 2).

Principal component analysis (PCA) showed that biological replicates within each time point clustered closely (Figure S2). The three developmental stages were clearly separated along PC1, which explained 52.09% of the total variance, while PC2 accounted for 12.51% of the variance. This indicated that the transcriptomic profiles of adventitious root formation in *C. lanceolata* were significantly distinct across the different stages. The good reproducibility within groups provided a reliable data foundation for subsequent identification of differentially expressed genes.

### 3.2. Functional Annotation

**Figure 1:**
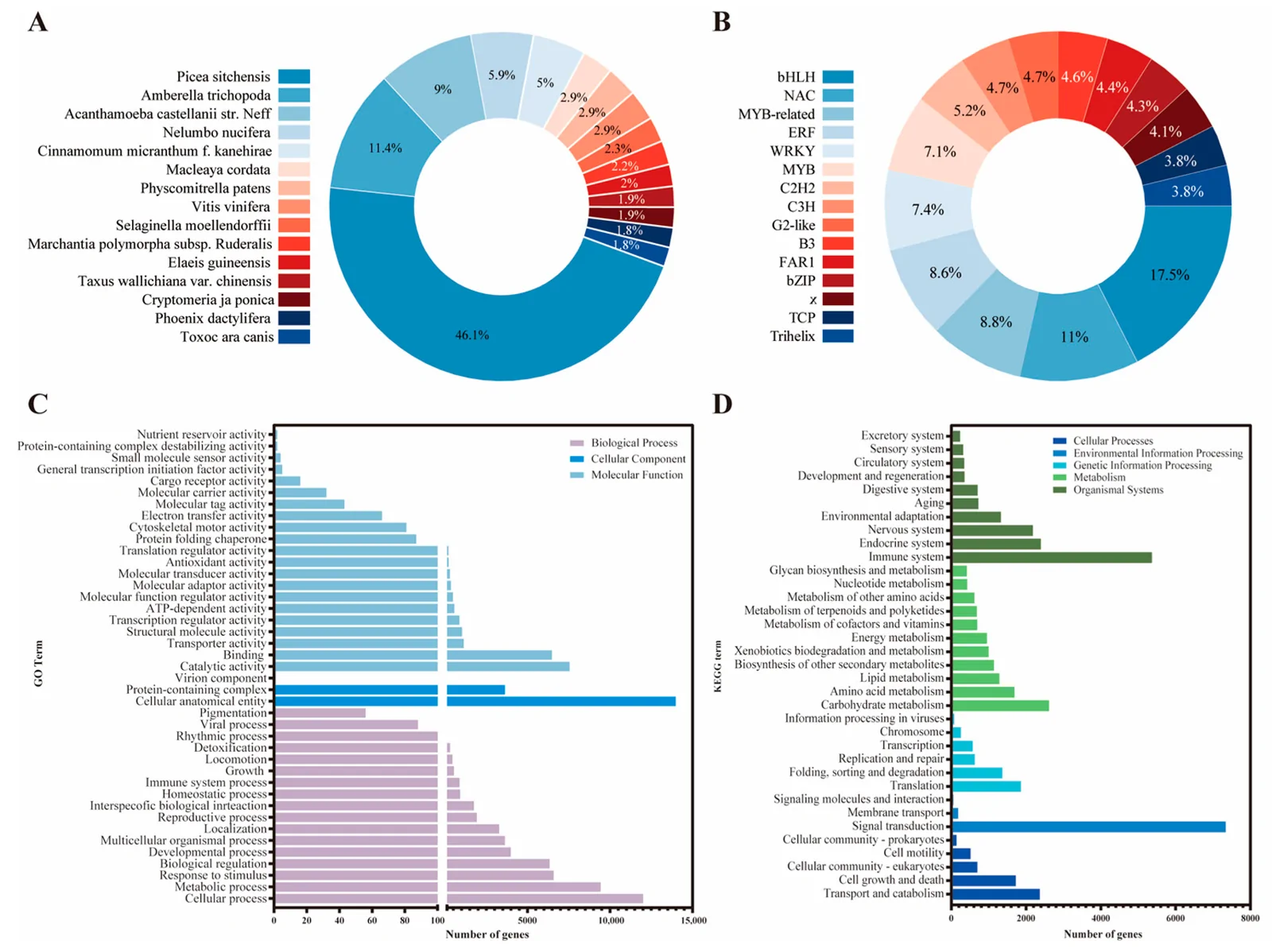
Functional annotation. (**A**) Species distribution of NR annotations; (**B**) distribution of transcription factor families; (**C**) database annotation statistics GO; (**D**) database annotation statistics KEGG.

To comprehensively obtain functional information of the genes, we aligned the 43,433 non-redundant Unigene sequences with coding potential against public databases, including NR, Swiss-Prot, KEGG, eggNOG, GO, and Pfam, using BLAST (v2.16.0) (E-value < 1 × 10^−5^). The annotation statistics (Figure S3) showed the following numbers and percentages of successfully annotated Unigenes in each database: Pfam (33,829, 77.9%), NR (32,886, 75.7%), Swiss-Prot (24,471, 56.3%), KEGG (21,905, 50.4%), eggNOG (21,086, 48.5%), and GO (15,932, 36.7%). Species distribution annotation based on the NR database (Figure 1A) revealed that *C. lanceolata* sequences showed the highest similarity to sequences from species such as spruce, accounting for 46.1%. At the level of regulatory elements, transcription factor analysis (Figure 1B) revealed that differentially expressed genes were significantly enriched in families including *bHLH*, *NAC*, *MYB*-related, *ERF*, *WRKY*, *MYB*, *C2H2*, and *C3H*. These transcription factors play central roles in plant growth, development, and signaling responses.

To further elucidate the biological pathways in which these genes are involved, we performed GO and KEGG functional classification. In the GO annotation (Figure 1C), Unigenes were mainly assigned to cellular processes and metabolic processes (biological process category), cellular anatomical entities and protein-containing complexes (cellular component category), and binding and catalytic activities (molecular function category). Notably, binding and catalytic activities are directly linked to the regulation of key enzymes and receptor proteins in phytohormone signal transduction. This provided a systematic classification basis for dissecting the key regulatory pathways underlying adventitious root development from a functional perspective. In addition, KEGG annotation (Figure 1D) showed that Unigenes were broadly distributed across five major categories: cellular processes, environmental information processing, genetic information processing, metabolism, and organismal systems. This comprehensive distribution indicates that the transcriptome data covered a wide range of biological functions.

### 3.3. Time-Series Analysis and KEGG/GO Enrichment of Differentially Expressed Genes

**Figure 2:**
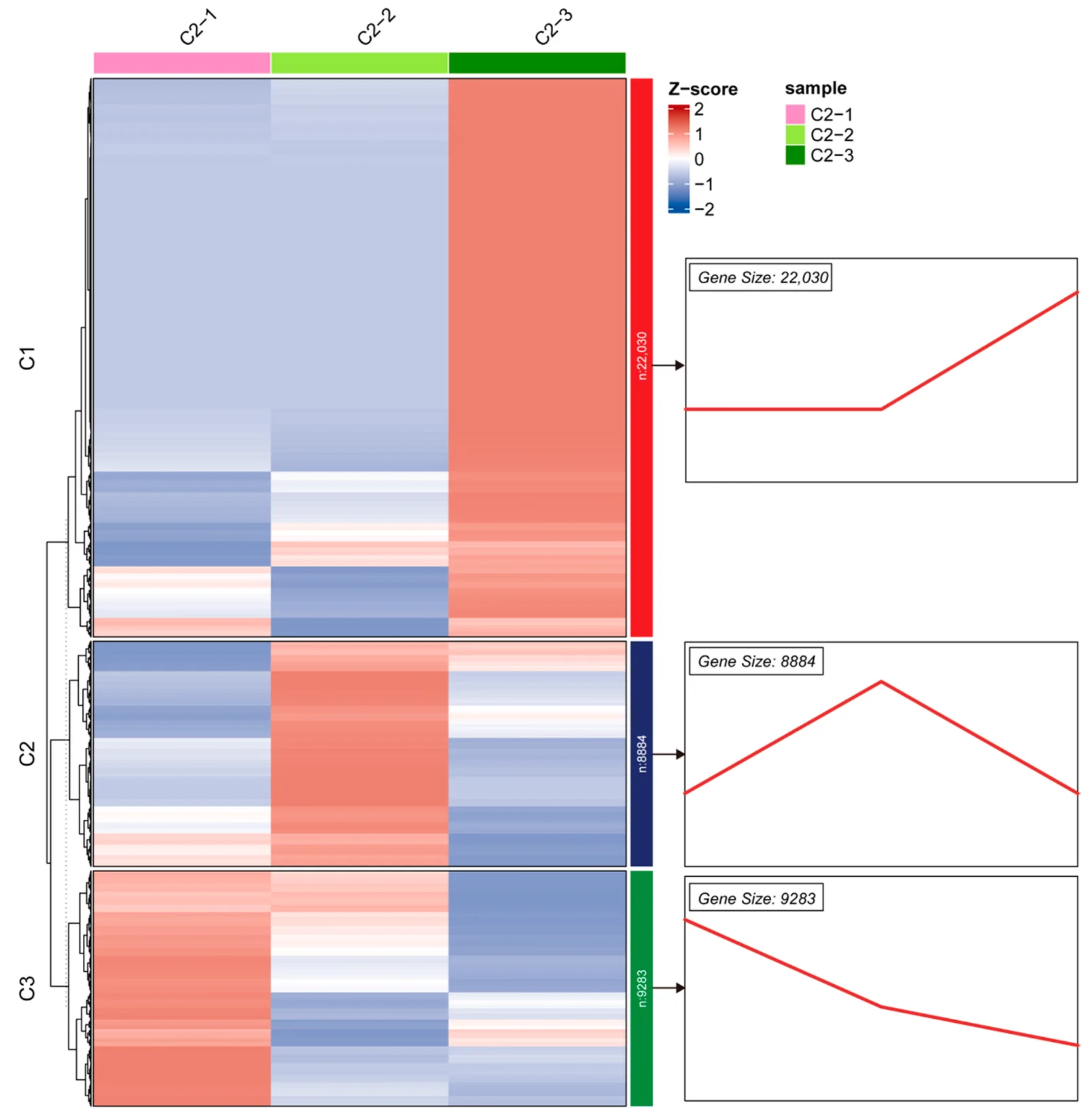
Analysis of time-series clustering.

Based on the functional annotations at the whole-transcriptome level, we further focused on identifying the key regulatory networks involved in adventitious root development in *C. lanceolata*. We analyzed the RNA-seq data from the three time points (7, 30, and 60 d after cutting). According to the dynamic expression patterns of the genes, we performed K-means clustering analysis on differentially expressed genes (DEGs) using their temporal expression profiles (Figure 2). The results showed that the DEGs were clearly divided into three clusters with distinct expression patterns. Cluster 1 contained 22,030 genes that were continuously upregulated throughout the developmental process. Cluster 2 contained 8884 genes whose expression reached a peak at 30 d and then plateaued or slightly declined. Cluster 3 contained 9283 genes that exhibited a continuous downregulation trend during development.

To further dissect the transcriptional changes between adjacent and non-adjacent developmental stages, we performed pairwise comparisons of the three time points and generated volcano plots (Figure S4). The pairwise comparisons revealed a marked increase in the number of differentially expressed genes over time: a total of 3627 DEGs were identified between 30 d and 7 d (C2-2 vs. C2-1), 10,422 DEGs between 60 d and 7 d (C2-3 vs. C2-1), and 10,666 DEGs between 60 d and 30 d (C2-3 vs. C2-2). This progressive increase in DEG numbers suggests that the transcriptional reprogramming during adventitious root formation becomes more pronounced as development proceeds, particularly during the later stages.

**Figure 3:**
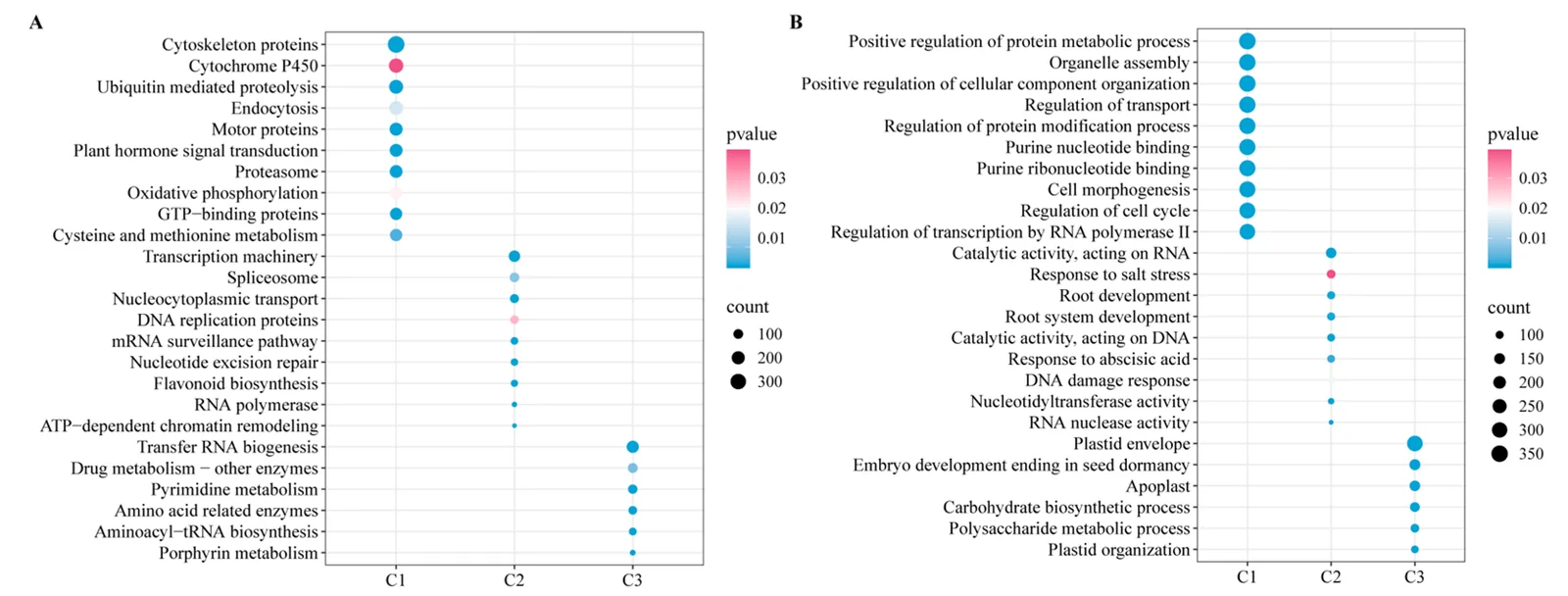
Comparison of the functional enrichment of three clusters with distinct expression patterns. (**A**) Functional enrichment analyses of GO; (**B**) functional enrichment analyses of KEGG. Enrichment was performed using the hypergeometric test with Benjamini–Hochberg correction (FDR < 0.05). Full statistical details, including adjusted *p*-values, enrichment ratios, and gene counts, are provided in Supplementary Tables S2 and S3.

To further explore the potential biological mechanisms underlying these clusters, we performed comparative functional enrichment analysis (Figure 3). The KEGG and GO enrichment results revealed that cluster 3 (early stage, 7 d) and cluster 2 (middle stage, 30 d) were mainly enriched in metabolism-related terms, such as pyrimidine metabolism (ko00240), carbohydrate biosynthetic process (GO:0016051), and nucleotidyltransferase activity (GO:0016779). In contrast, cluster 1 (late stage, 60 d) was significantly enriched in signal transduction-related terms, including phytohormone signal transduction (ko04075), motor proteins (ko04814), and regulation of transport (GO:0032409). The complete lists of significantly enriched KEGG pathways and GO terms are provided in Tables S2 and S3. These results indicate that adventitious root formation in *C. lanceolata* cuttings exhibits a temporal functional shift, from early metabolic reprogramming to late dominance by signal transduction networks.

Notably, although active metabolic networks provide nucleic acid precursors and carbon skeletons for cell division and proliferation of root primordia at early stages, phytohormones act as the upstream central hub governing the entire rooting process. They directly orchestrate and reprogram peripheral metabolic networks—such as pyrimidine and carbohydrate metabolism—by activating downstream transcriptional cascades. Previous studies have confirmed that phytohormone signal transduction is a core regulatory pathway in adventitious root formation of woody plants during cutting propagation. Therefore, given the decisive regulatory role of this pathway in the adventitious root development network of *C. lanceolata*, we will further focus on and deeply explore the phytohormone signal transduction pathway, aiming to systematically elucidate its molecular regulatory mechanisms.

### 3.4. Expression Analysis of Pathways

**Figure 4:**
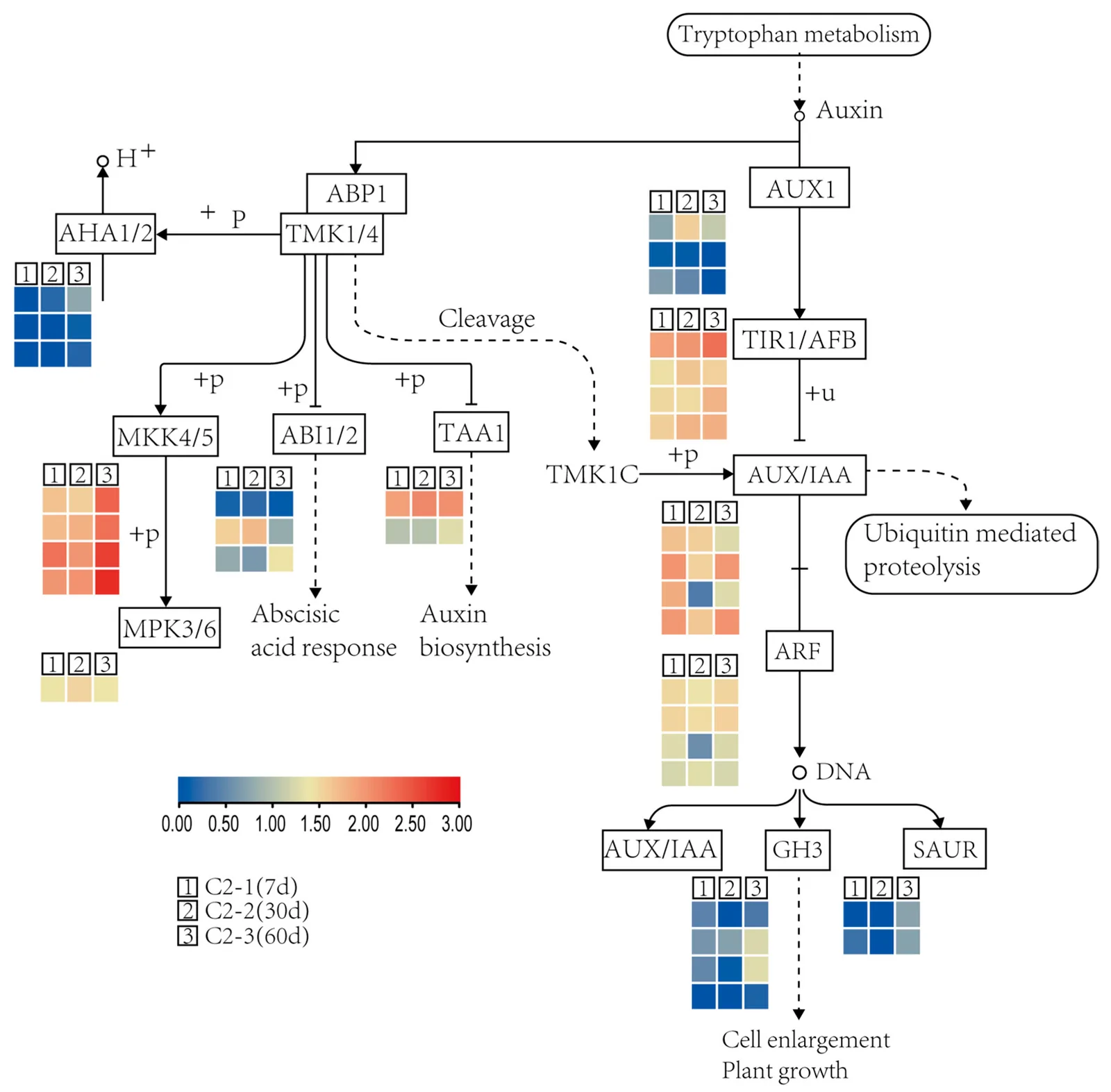
Schematic of the plant hormone signal transduction pathway involved in the growth and development of *C. lanceolata*. 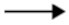: activation; 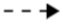: indirect effect; 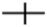: dissociation; 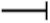: inhibition.

The enrichment analysis above established the central regulatory role of phytohormone signal transduction in the rooting process. To further dissect the internal architecture and dynamic activation pattern of this network during adventitious root formation in *C. lanceolata*, we mapped the relevant differentially expressed genes (DEGs) onto the KEGG pathway framework (Figure 4). The results showed that multiple key components of the auxin signaling pathway, including *AUX1* (auxin influx carrier), *TIR1/AFB* (auxin receptors), *Aux/IAA* (transcriptional repressors), and *GH3/SAUR* (downstream response genes), exhibited significant differential expression patterns. The coordinated expression patterns of these genes suggest their potential involvement in the initiation and development of adventitious root primordia.

In this process, ubiquitin-mediated proteolysis served as a core node connecting *Aux/IAA* degradation and *ARF* activation. It was continuously activated, thereby relieving the transcriptional repression of *ARF* by *Aux/IAA* and successfully initiating the expression of rooting-related downstream genes. Additionally, components of the ABA signaling pathway (ABI1/2) engaged in critical crosstalk with auxin signaling through the MKK4/5-MPK3/6 module. The shared pathway architecture of this module suggested that ABA signaling may modulate the output of auxin signaling at specific developmental stages of adventitious root formation, ultimately facilitating cell expansion and root growth phenotypes. This pathway-level analysis down to individual gene nodes not only systematically validated the global enrichment results above, but also further highlighted the potential importance of auxin signaling and transport during the rooting process.

### 3.5. Identification, Phylogeny, and Expression Analysis of the PIN Gene Family

**Figure 5:**
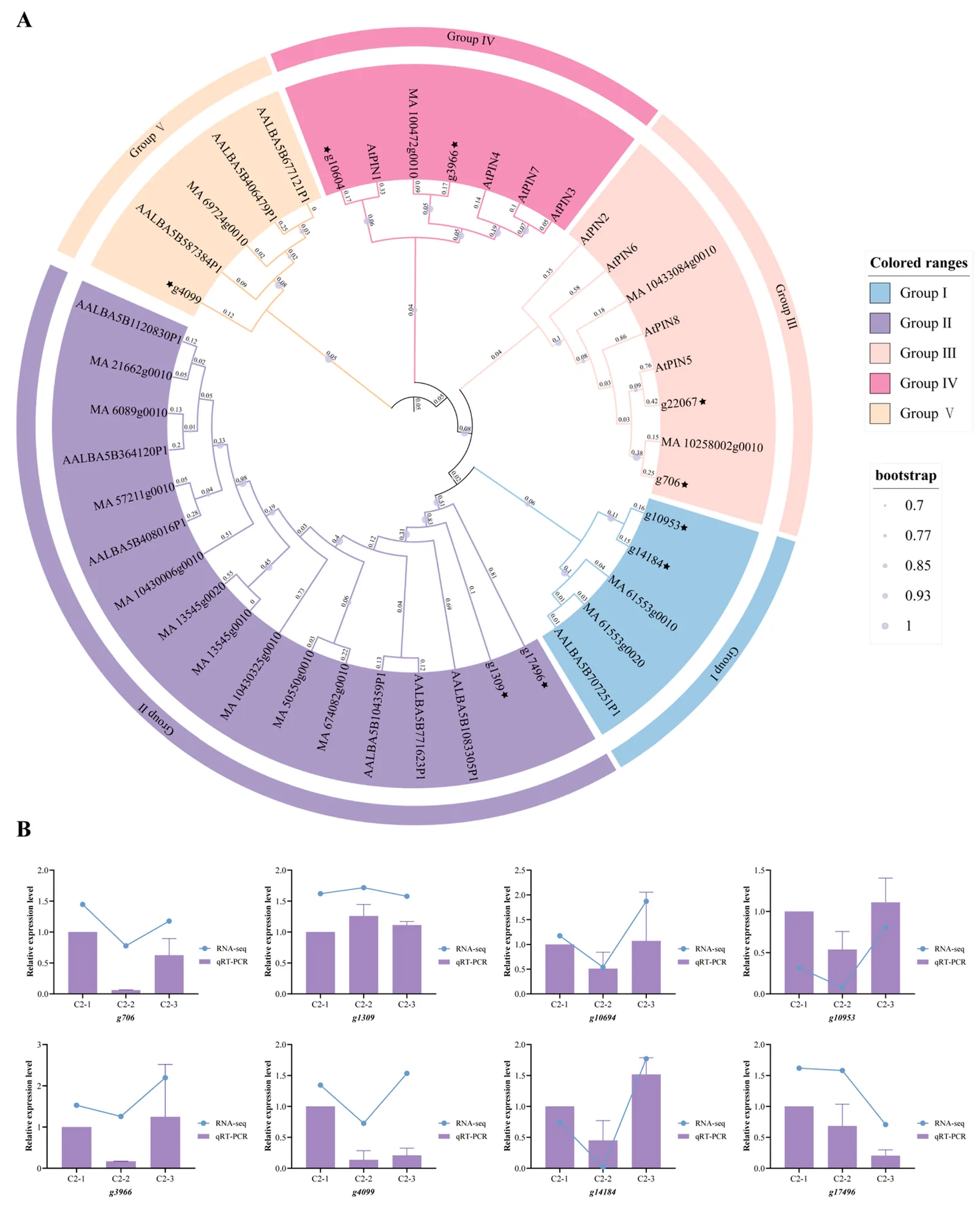
Expression analysis of the *PIN* gene family. (**A**) Phylogenetic analysis of *PIN*s from *A. thaliana*, *C. lanceolata*, *P. abies and A. alba*; ★ represent *C. lanceolata PIN* genes; (**B**) comparison of expression trends between candidate gene RNA-seq and qRT-PCR.

As core auxin efflux carriers, *PIN* family proteins are positioned at the upstream input layer of this signaling transduction network. They mediate directional cell-to-cell auxin transport, establishing local concentration gradients and auxin maxima. This provides the spatial prerequisite for signal perception by downstream *TIR1/AFB* receptors. However, the molecular characteristics of *PIN* family members in *C. lanceolata* and their expression patterns during adventitious root formation remain unclear. In this study, we identified nine *PIN* gene family members in the *C. lanceolata* transcriptome by combining HMMER domain searches and BLASTP homology searches. The phylogenetic tree constructed with *A. thaliana*, *Picea abies* (*P. abies*) and *Abies alba Mill* (*A. alba*) PIN proteins (Figure 5A) showed that these *C. lanceolata PIN* genes were classified into five subgroups, with members distributed across all major clades. This suggests that they may have undergone extensive functional divergence during conifer evolution and development.

To further characterize the nine identified PIN proteins, we analyzed their physicochemical properties, transmembrane topology, conserved motifs, and subcellular localization. The protein length ranged from 403 to 670 amino acids, with molecular weights ranging from 43.7 to 73.5 kDa and theoretical isoelectric points (pIs) from 6.42 to 9.42 (Table S4). Transmembrane domain prediction using TMHMM revealed variations in the number of transmembrane helices among members, with seven proteins containing 10 transmembrane helices (Table S6). Conserved motif analysis using the MEME Suite identified 10 conserved motifs (Figure S5). Subcellular localization prediction was performed using WoLF PSORT. Subcellular localization prediction assigned nine PIN proteins to the plasma membrane as the primary destination, with varying secondary localizations among members (Table S5).

To investigate the specific roles of these *PIN* genes in adventitious root development in *C. lanceolata*, we analyzed their temporal expression patterns across the three developmental stages (Figure S6). The results revealed that different *PIN* genes exhibited markedly divergent expression trends. Among them, several *PIN* genes showed significantly lower expression at 30 d (C2-2) compared to 7 d (C2-1) and then rebounded to higher levels at 60 d (C2-3). Other members displayed either continuous downregulation or an initial upregulation followed by downregulation. This complex temporal expression pattern indicates that the auxin signaling pathway may dynamically mediate polar auxin transport by fine-tuning the transcription of different *PIN* family members, which may contribute to adventitious root initiation and elongation in *C. lanceolata*.

To further validate the reliability of the transcriptomic expression profiles, we selected nine candidate *PIN* genes for qRT-PCR validation, using *C. lanceolata* Actin as the internal reference gene (primers listed in Table S1). Melting-curve analysis confirmed the specificity of the primer pairs (Figure S7). Comparative analysis showed that the relative expression trends detected by qRT-PCR were highly consistent with the RNA-seq data (Figure 5B), confirming both the accuracy of the transcriptome data and the reliability of the expression analysis results.

## 4. Discussion

*C. lanceolata* is one of the most important timber tree species in southern China. However, the molecular regulatory mechanisms governing adventitious root formation during cutting propagation of *C. lanceolata* remain incompletely understood. The lack of genetic resources has severely hindered molecular breeding efforts in this species. In this study, we performed time-series transcriptome sequencing of roots from the elite *C. lanceolata* clone ‘Minshan No. 1’ at three critical time points [11,12]. We obtained high-quality transcriptomic data and identified members of the *PIN* gene family. This provides preliminary evidence that polar auxin transport might contribute to adventitious root formation in *C. lanceolata*.

A total of approximately 69.30 Gb of clean data were generated by Illumina sequencing. De novo assembly and gene prediction yielded 43,433 coding genes, of which 32,886 (75.7%) were successfully functionally annotated. This data scale is comparable to that of transcriptomic studies on somatic embryogenesis in the conifer *Picea abies* [26]. This study represents the first establishment of a time-series transcriptomic profile for *C. lanceolata* under the specific context of adventitious root formation during cutting propagation. Compared with transcriptomic studies of adventitious root development in *Citrus* spp. [27], our dataset shows greater gene coverage. This may be attributed to the larger genome and abundant gene family members of *C. lanceolata* as a gymnosperm [28,29].

Temporal clustering and comparative functional enrichment analyses revealed a critical temporal shift in functional profiles. Early stages of adventitious root development in *C. lanceolata* were dominated by active metabolic processes, such as pyrimidine metabolism (ko00240) and carbohydrate biosynthesis (GO:0016051). In contrast, later stages were significantly enriched in phytohormone signal transduction (ko04075). This temporal pattern is highly consistent with transcriptomic features observed during adventitious root formation in green cuttings of *Prunus persica* [30]. A similar phenomenon was also reported in studies on adventitious root formation in *Metasequoia glyptostroboides* [31]. Notably, the early enrichment in pyrimidine metabolism and carbohydrate biosynthesis likely corresponds to the intense cell division and energy metabolism required for the initiation of adventitious root primordia [32]. This finding anchors the metabolic reprogramming events underlying early primordium formation to a specific temporal window. The later enrichment in phytohormone signal transduction points to a sustained dominant role of auxin signaling during the elongation and maintenance phases of adventitious rooting.

Within the phytohormone signal transduction network, we identified multiple key components of the auxin signaling pathway that exhibited significant differential expression patterns. Specifically, the homologous gene of the auxin influx carrier *AUX1* showed relatively high expression at 7 d, suggesting that polar auxin transport was initiated at the early stage of adventitious root formation. Members of the auxin receptor *TIR1/AFB* family reached peak expression at 30 d, which is similar to the temporal activation pattern of *TIR1/AFB* during lateral root initiation in *A. thaliana* [33,34]. Multiple members of the *Aux/IAA* transcriptional repressor family showed continuous downregulation from 7 d to 30 d, whereas downstream response genes such as *SAUR* and *GH3* were significantly upregulated at 60 d. This expression profile is consistent with the activation of an auxin signaling cascade at the transcriptional level during adventitious root formation in *C. lanceolata*. Its dynamic activation window parallels the temporal expression patterns of *MdPINs* and *MdARFs* during adventitious root development in *M. domestica* [9]. Additionally, we found that the ABA signaling components *ABI1/2* engaged in critical crosstalk with auxin signaling through the MKK4/5-MPK3/6 module (Figure 4). This is consistent with findings in *Cinnamomum kanehirae*, where auxin and abscisic acid were identified as core regulators of adventitious root formation [35]. This suggests that ABA signaling may finely modulate the output intensity of auxin signaling at specific developmental stages of adventitious root formation, thereby coordinating cell expansion and the macroscopic phenotype of root growth.

Furthermore, during the rooting process of *C. lanceolata* cuttings, endogenous IAA content gradually increases and reaches its peak at the stage of adventitious root formation, whereas ABA and ZT contents decline continuously, suggesting that the elevation of the IAA/ZT ratio may create favorable conditions for root primordium initiation [36]. In *P. abies*, ethylene promotes rooting by accelerating the breakdown of cytokinins [37]; jasmonic acid, acting as a wound signal, can promote IAA synthesis through ERF109, thereby coupling cutting-induced wounding to auxin signaling [38]. In addition, gibberellin generally inhibits adventitious root formation, while abscisic acid exhibits concentration-dependent dual effects [39]. The synergistic and antagonistic interactions among these hormones collectively constitute a complex regulatory network governing adventitious root formation [39].

To elucidate the evolutionary relationships and functional implications of the *PIN* gene family in *C. lanceolata*, we constructed a phylogenetic tree incorporating the nine PIN proteins identified from *C. lanceolata* along with *PIN* homologs from *A. thaliana*, *P. abies*, and *A. alba*. The resulting topology resolved all sequences into five major subgroups, with the nine *C. lanceolata PIN* members distributed across each of these clades, suggesting that the *PIN* family in this species has undergone considerable functional diversification over evolutionary time. Intriguingly, subgroup II was composed exclusively of gymnosperm members and lacked any *A. thaliana* counterparts, implying that this clade may represent a conifer-specific *PIN* lineage that could be associated with unique developmental processes in gymnosperm processes that are not well represented in *A. thaliana*. In contrast, several *C. lanceolata* PIN proteins clustered with functionally well-characterized *A. thaliana PIN*s within the remaining subgroups, indicating that these *C. lanceolata PIN* genes are evolutionarily conserved and likely perform analogous functions in mediating polar auxin efflux and establishing local auxin gradients. Collectively, our phylogenetic analysis reveals both conserved and potentially species-specific functional branches within the *C. lanceolata PIN* family, providing an evolutionary framework for further functional dissection of individual *PIN* members in adventitious root formation.

The systematic identification and expression analysis of the *PIN* gene family further established its regulatory role at the upstream input layer of auxin signaling. In this study, we identified nine *PIN* gene family members in the *C. lanceolata* transcriptome, and phylogenetic analysis revealed their distribution across four subgroups. Transcriptomic data showed that *C. lanceolata PIN* genes exhibited differential expression trends across the three time points of adventitious root development. This dynamic expression pattern is consistent with the differential expression characteristics of the *MdPIN* family genes throughout adventitious root development in *M. domestica*. Specifically, *MdPIN8* and *MdPIN10* were upregulated during the induction stage, all *MdPINs* were upregulated during the initiation stage, and *MdPIN4*, *MdPIN5*, and *MdPIN8* were upregulated during the elongation stage [9]. In *Olea europaea*, *OePIN* genes also exhibited differential expression during IBA-induced adventitious root formation, and wound-related reactive oxygen species may influence auxin transport by regulating *OePIN* expression [40]. Our results further support the functional model in which PIN proteins may participate in directional cell-to-cell transport and establish local auxin concentration gradients, thereby providing the spatial prerequisite for signal perception by downstream *TIR1*/*AFB* receptors.

This study has certain limitations. First, our *PIN* identification was based on transcriptomic data, which only reflect expression under specific tissues, stages, or treatments, and thus may not cover all members, especially those with very low abundance or restricted spatiotemporal expression. This limitation was unavoidable as the *C. lanceolata* genome was unavailable at the time. As genomic data improve, future genome-level identification and functional annotation will be needed to validate and extend our preliminary findings. Second, a major bottleneck in functionally validating these *PIN* genes is the lack of a reliable genetic transformation platform in our study material; consequently, we are unable to perform stable overexpression or knockout experiments, and thus their physiological roles remain to be confirmed once such a system becomes available. Third, the sampling time intervals were relatively large; future studies could use shorter intervals to reveal the early molecular dynamics of adventitious root formation. Fourth, we acknowledge that the absence of histological sectioning and quantitative phenotypic data (e.g., rooting percentage, root length, or biomass) in the present study limits our ability to precisely assign the three sampling time points to definitive stages of adventitious root formation. In future work, we plan to incorporate fine-scale time-series sampling, histological observations, and rooting performance assays to better anchor transcriptomic changes to discrete developmental events and to functionally validate the candidate genes identified here.

In summary, through transcriptome sequencing, comparative functional enrichment analysis, and identification of the *PIN* gene family, this study systematically revealed a temporal regulatory landscape during adventitious root formation in *C. lanceolata* cuttings, characterized by a shift from early metabolic reprogramming to late phytohormone signal transduction. We identified the dynamic expression patterns of key components in the auxin signaling pathway. We also identified nine *PIN* gene family members in *C. lanceolata* and characterized their differential expression patterns during adventitious root development. These findings enrich the genetic information resources for *C. lanceolata* and provide a transcriptomic basis for understanding the molecular mechanisms of adventitious root formation in conifer cuttings.

## 5. Conclusions

In this study, we performed time-series transcriptome sequencing at different developmental stages (7, 30, and 60 d) of adventitious root formation in cuttings of elite *C. lanceolata* clones. We successfully assembled and annotated a large number of high-quality gene sequences, greatly enriching the genomic and transcriptomic resources for *C. lanceolata*. Our results systematically revealed a distinct temporal functional transition during adventitious root development, shifting from early metabolic reprogramming (e.g., pyrimidine and carbohydrate metabolism) to late dominance by phytohormone signal transduction networks. During this process, the auxin signaling pathway may function as an important regulatory hub during root growth and development. On this basis, we further identified nine members of the *PIN* gene family at the whole-transcriptome level in *C. lanceolata*. Both the temporal expression profiles and qRT-PCR validation consistently indicated that the dynamic differential expression of *PIN* genes may contribute to the initiation and elongation of adventitious root primordia by finely regulating polar auxin transport. In summary, this study provides transcriptomic insights into the molecular regulatory network associated with adventitious root formation in *C. lanceolata* cuttings, but also provides important theoretical basis and candidate gene targets for efficient propagation and molecular improvement of elite *C. lanceolata* clones.

## Figures and Tables

**Table 1:** The analysis of data output quality.

Samples	Total Reads	Clean Reads	Percentage of Clean Reads	Total Bases	Clean Bases	GC Content	Q20	Q30
C2-1-1	39,954,166	39,954,166	100.00%	5,993,124,900	5,951,798,696	43.38%	99.39%	98.03%
C2-1-2	47,034,140	47,034,140	100.00%	7,055,121,000	7,026,170,428	43.47%	99.43%	98.15%
C2-1-3	41,554,000	41,554,000	100.00%	6,233,100,000	6,202,876,730	43.24%	99.38%	98.04%
C2-2-1	44,816,882	44,816,882	100.00%	6,722,532,300	6,695,666,320	42.94%	99.42%	98.10%
C2-2-2	46,755,474	46,755,474	100.00%	7,013,321,100	6,965,855,608	42.91%	99.41%	98.15%
C2-2-3	54,198,594	54,198,594	100.00%	8,129,789,100	8,081,850,938	43.11%	99.34%	97.88%
C2-3-1	64,869,800	64,869,800	100.00%	9,730,470,000	9,693,784,068	43.33%	99.48%	98.13%
C2-3-2	59,573,194	59,573,194	100.00%	8,935,979,100	8,908,297,438	43.24%	99.48%	98.13%
C2-3-3	66,105,062	66,105,062	100.00%	9,915,759,300	9,861,644,746	43.32%	99.46%	98.07%

**Table 2:** Assembly result statistics.

Transcript Length Interval	200–500 bp	500–1000 bp	1000–2000 bp	>2000 bp	Total
Number of transcripts	342,397	79,996	59,406	60,362	542,161
Number of unigenes	342,358	73,287	31,092	22,996	469,733
Number of coding genes	3011	11,439	13,810	15,173	43,433

## Data Availability

The original contributions presented in this study are included in the article/Supplementary Material. Further inquiries can be directed to the corresponding authors.

## Supplementary Materials

The following supporting information can be downloaded at https://www.mdpi.com/article/10.3390/genes17091008/s1, Figure S1. Phenotypes of *C. lanceolata* cuttings at 7, 30 and 60 d after cutting. (A) Cuttings at 7 d, (B) cuttings at 30 d, (C) cuttings at 60 d; Figure S2: Principal component analysis of *C. lanceolata* samples; Figure S3: Upset plot of functional annotation statistics across six public databases; Figure S4. Time-course differential expression analysis of adventitious root formation in *C. lanceolata* cuttings. (A) C2-2VSC2-1, (B) C2-3VSC2-1, (C) C2-3VSC2-2; Figure S5. Conserved motif analysis of PIN proteins in *C. lanceolata*; Figure S6: Heatmap analysis of *PINs* expression in the growth and development of *C. lanceolata*; Figure S7: Melting curves for qRT-PCR primers; Table S1: List of the primers used for qRT-PCR; Table S2: KEGG enrichment results; Table S3: GO enrichment results; Table S4: Physicochemical properties of the *PIN* gene family proteins in *C. lanceolata*; Table S5: Prediction of *PIN* gene subcellular localization; Table S6: Transmembrane topology of PIN proteins in *C. lanceolata*.
